# Epidermal Growth Factor Stimulates Transforming Growth Factor-Beta Receptor Type II Expression In Corneal Epithelial Cells

**DOI:** 10.1038/s41598-019-42969-2

**Published:** 2019-05-30

**Authors:** Daisy Y. Shu, Audrey E. K. Hutcheon, James D. Zieske, Xiaoqing Guo

**Affiliations:** 1 Schepens Eye Research Institute/Massachusetts Eye and Ear and Department of Ophthalmology, Harvard Medical School, 20 Staniford Street, Boston, MA 02114 USA; 2 Save Sight Institute, Discipline of Ophthalmology, Sydney Medical School, The University of Sydney, 8 Macquarie St, Sydney, NSW 2000 Australia

**Keywords:** Cell culture, Reverse transcription polymerase chain reaction, Fluorescence imaging, Western blot, Growth factor signalling

## Abstract

We previously demonstrated that inhibition of epidermal growth factor receptor (EGFR) slowed corneal epithelial migration. Here we examine the effect of EGF on transforming growth factor-beta receptor II (TGF-βRII) in a corneal wound-healing model and primary human corneal epithelial cells (pHCE). Corneal debridement wounds were made and allowed to heal ± Tyrphostin AG1478 (EGFR inhibitor), and assayed for EGFR activation and EGFR and TGF-βRII localization. Primary HCE were treated with EGF ± U0126 (MEK inhibitor) and assayed for TGF-βRII expression. EGFR activation was maximal 15 minutes after wounding and localized in the migrating epithelial cells. TGF-βRII localization was also observed in the migrating epithelium and was reduced when EGFR was blocked. When pHCE were treated with EGF for 6 hours, the cells produced enhanced levels of TGF-βRII, which was blocked by U0126. Downstream signaling pathways of MEK (p38^MAPK^ and ERK1/2^MAPK^) were then examined, and TGF-β1 and EGF were found to have differential effects on the phosphorylation of p38 and ERK1/2, with TGF-β1 upregulating p-p38 but not pERK1/2 and EGF upregulating pERK1/2 but not p-p38. Taken together, these data indicate that EGF stimulates TGF-βRII through ERK1/2 and EGFR signaling, suggesting interplay between EGF- and TGF-β-signaling pathways during corneal wound repair.

## Introduction

Corneal epithelial wound healing is a complex and ordered process that involves epithelial cell proliferation, migration, adhesion, and differentiation. This process is regulated in part by a number of soluble growth factors—epidermal growth factor (EGF) family, transforming growth factor beta (TGFβ) family, hepatocyte growth factor (HGF), fibroblast growth factor (FGF), and platelet derived growth factor (PDGF)^1–3^.

Among these growth factors is EGF, which acts on a cell by binding with high affinity to the EGF receptor (EGFR) on the cell’s surface, thus stimulating the ligand-induced receptor dimerization^4^ and activating the intrinsic protein-tyrosine kinase activity of the receptor^5,6^. This tyrosine kinase activity further initiates signaling cascades, resulting in a variety of biochemical changes within the cell—rapid alterations in intracellular calcium levels, increased glycolysis and protein synthesis, activation of gene transcription, receptor down-regulation, and ultimately, stimulating DNA synthesis and cell proliferation^5–9^. Numerous studies have shown that EGF participates in dermal wound healing and accelerates wound closure through stimulation, proliferation, and migration of keratinocytes, endothelial cells, and fibroblasts, thus facilitating dermal regeneration^10–14^. Furthermore, clinical trials and animal studies have proven that the addition of topical EGF enhances corneal epithelial wound healing^15–17^.

Another growth factor family that influences wound healing is the TGFβ family. TGFβ is a multifunctional cytokine that has a broad spectrum of actions, affecting all cell types and is involved in all stages of wound healing^1,18–22^. In mammals, TGFβ exists in three isoforms (TGF-β1, -β2, and -β3) that all bind to a specific transmembrane receptor, TGF-βRII. TGF-βRII then combines with and activates TGF-βRI, which in turn exerts its biological effects through different signaling pathways—the canonical Smad-signaling pathway and non-canonical pathways, including p38^MAPK^ signaling^23,24^.

EGF and its associated downstream signaling pathways have been shown to modulate TGFβ signaling in different cell types^25–29^. For example, Dunfield and Nachtigal reported that in primary human ovarian cancer cells, EGF decreased the TGFβ-induced mRNA expression of the cell cycle regulator, p15^INK4B^, thus decreasing the sensitivity of the ovarian cancer cells to the anti-proliferative effect of TGFβ^25^. In addition to activating EGFR-signaling, EGF is known to activate several signaling pathways including the Ras-Raf-ERK1/2^MAPK^, phospholipase C gamma protein, Jak/STAT, JNK and PI3K/Akt^30^. Oncogenic Ras inhibited TGFβ signaling in mammary and lung epithelial cells by negatively regulating the TGFβ mediators of Smad2 and Smad3^27^. In addition, the EGF-ERK pathway positively regulated Smad2 signaling in COS7 cells by phosphorylating Smad2^31^, and INFγ upregulated the inhibitory Smad, Smad7, by acting through Jak1 and Stat1, thereby preventing the interaction between Smad3 and the TGFβ receptor^28^. Finally, the activation of ERK-MAPK by HGF and EGF was shown to inhibit the translocation of Smads to the nucleus^32,33^. Interestingly, in murine embryonic maxillary mesenchymal cells, EGF and TGFβ-signaling pathways do not converge^34^.

Previously, we observed that TGF-βRII was upregulated in response to corneal epithelial wounding^22^, and in a separate study, we showed that the inhibition of EGFR slowed epithelial migration rates^35^. In addition, we found that TGFβ1 upregulated p15 expression in corneal epithelial cells, which resulted in increased cell migration^36^. These findings raised the following question: Do the EGF- and TGFβ-signaling pathways converge in corneal epithelial cells? Therefore, in the present study, we examined if EGF modulated the TGFβ-signaling pathway by stimulating the synthesis of TGF-βRII at both the mRNA and protein level in corneal epithelial tissue.

## Results

### Activation of EGFR in the wounded corneal epithelium

**Figure 1:**
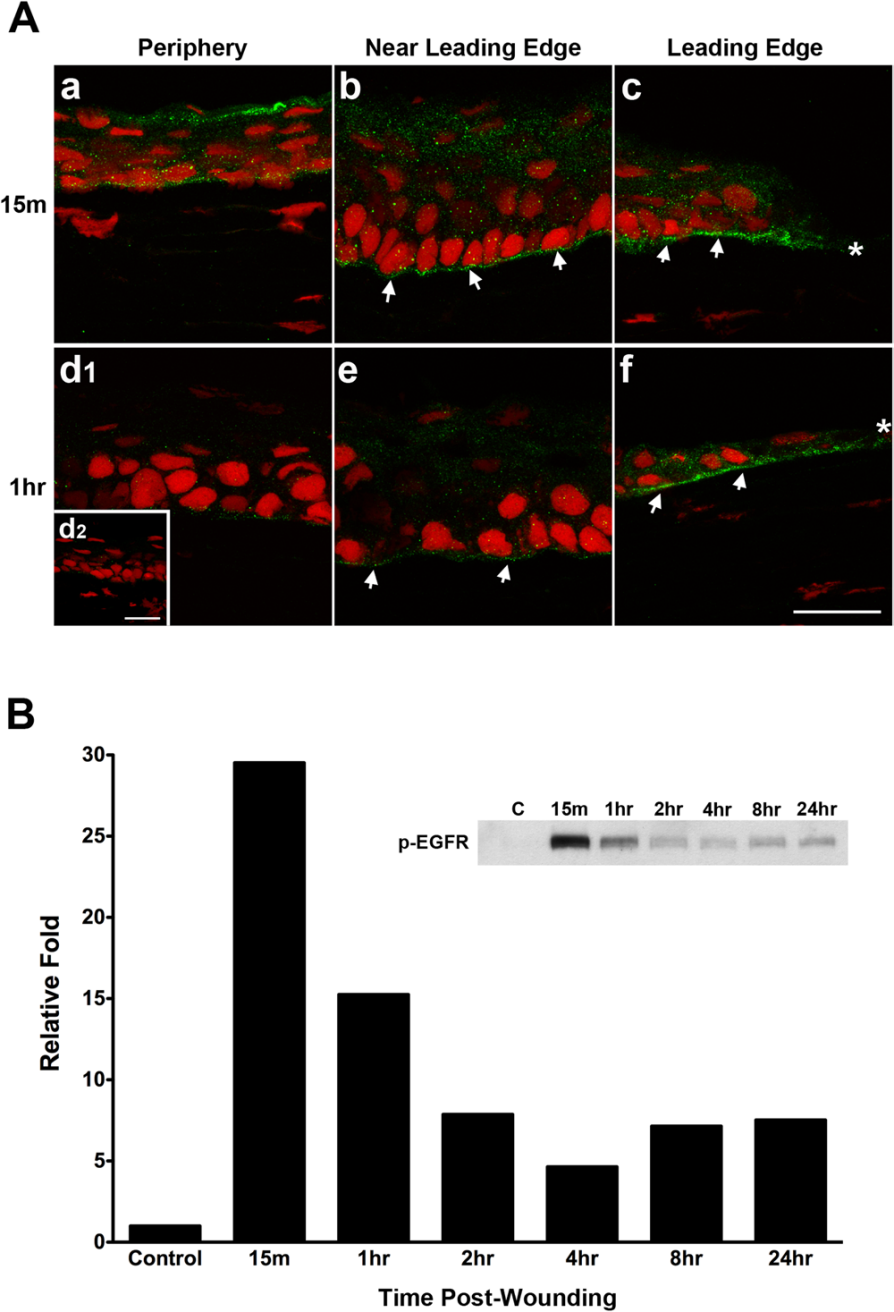
Activation of EGFR in the wounded corneal epithelium. (**A**) Representative indirect-immunofluorescent (IF) images of p-EGFR localization (green) following a 3 mm debridement (**A**.a–c) 15 minutes (15 m) and (**A**.d–f) 1 hour (1 hr) after wounding. As soon as 15 m, p-EGFR was upregulated at the tip of the leading edge (**A**.c; *), localizing mainly at the basal epithelial aspect adjacent to the basement membrane (**A**.b,c; arrows). Localization of p-EGFR was observed all the way out to the peripheral epithelium (**A**.a). By 1 hr, this localization decreased in the peripheral region (**A**.d1), but continued near and at the leading edge (**A**.g,f; arrows). No obvious activation of EGFR was observed in the unwounded rat corneal epithelium (**A**.d2). Propidium Iodide (red) counterstained for all cell nuclei. Scale bar = 25 μm. (**B**) In agreement with the IF data, western blot of the wounded corneal epithelium showed a peak of p-EGFR at 15 m post-wounding and then a decrease in EGFR activation over time. Interestingly, p-EGFR did not return to Control levels by 24 hours (24 hr). Data is from a pooled sample of 10 animals and reported as relative fold with samples normalized to Control (no treatment), which was set to 1. Original western blot presented is available in Supplemental Fig. S1.

We, as well as others, have demonstrated that EGFR is activated during corneal wound repair^2,35,37–40^, and this activation was found to be due to the phosphorylation of total EGFR rather than the increase of gene or protein expression^35^. In this series of experiments, we confirmed EGFR activation by observing phospho (p)-EGFR localization and expression in corneal epithelium after 3 mm debridement wounds, which were allowed to heal *in vivo* up to 24 hours. As seen in Fig. 1A.a–c, EGFR was activated in the epithelium as early as 15 minutes (15 m) post-wounding, and was most prominent at the leading edge (Fig. 1A.c; *indicates the tip of the leading edge) and immediately adjacent to the leading edge (near leading edge: Fig. 1A.b), especially in the basal epithelial aspect adjacent to the basement membrane (Fig. 1A.b,c; arrows). Localization of p-EGFR extended all the way out to the periphery of the cornea (Fig. 1A.a). At one hour (1 hr) post-wounding (Fig. 1A.d–f), p-EGFR was preferentially localized at the epithelial leading edge (Fig. 1A.f, *indicates tip of leading edge) along the basal aspect of the epithelium adjacent to the basement membrane (Fig. 1A.f, arrows). Faint p-EGFR was present near the leading edge (Fig. 1A.e; arrows) and little, if any, was present in the peripheral cornea (Fig. 1A.d1). No p-EGFR localization was observed in the unwounded rat corneal epithelium (Fig. 1A.d2). Western blot result (Fig. 1B) confirmed that the peak of p-EGFR in the corneal epithelium was at 15 minutes post-wounding. At this time point, p-EGFR was upregulated by about 30-fold compared to unwounded control (C). One hour after wounding, p-EGFR decreased by about half. The p-EGFR protein levels remained elevated in wounded corneas compared to unwounded corneal epithelium for at least 24 hours, with protein levels decreasing by about 50% each hour until 4hrs. At 8 hrs, the protein level increases slightly and appears to plateau. Graphical data represents corneal epithelial samples from 10 animals that were pooled together and normalized to unwounded control (set to 1). Images in Fig. 1A are representative of at least 3 animals per time point.

### Inhibition of EGFR activation in the wounded cornea

**Figure 2:**
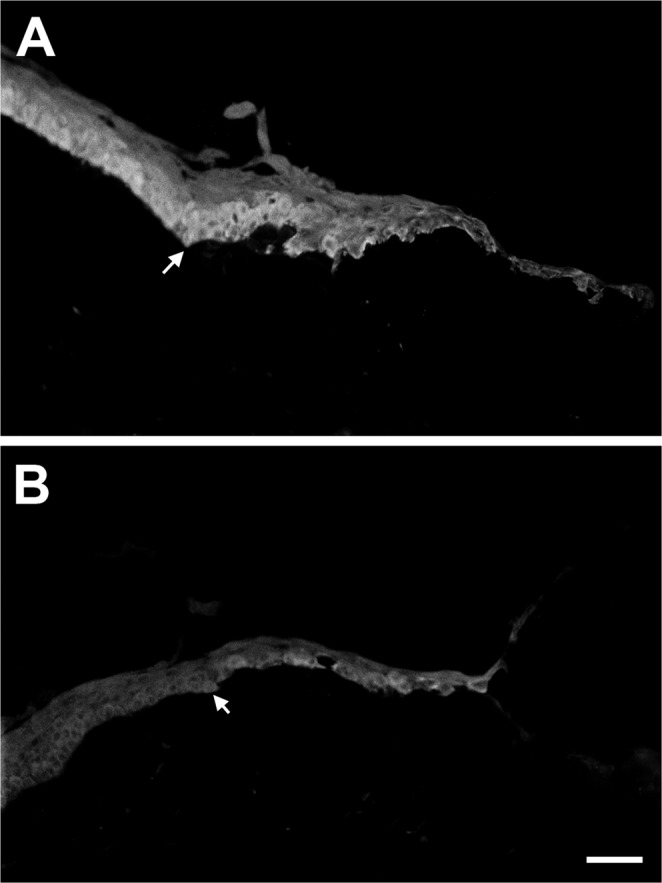
Inhibition of EGFR activation in the wounded cornea. Representative indirect-immunofluorescent (IF) images of TGF-βRII localization in rat corneal epithelium following a 3 mm debridement and treated for 18 hours without (**A**) and with (**B**) Tyrphostin AG1478 (AG1478). In untreated corneas (**A**), TGF-βRII was localized and upregulated in migrating cells near the leading edge; however, with the addition of AG1478 (**B**), TGF-βRII localization was decreased in these cells. Arrow indicates wound edge. Scale bar = 50 μm.

Previously, we reported that TGF-βRII was present at low levels in the central cornea of unwounded rat corneal epithelium and much higher levels in the limbus^22^. After wounding, the level of TGF-βRII increased in the epithelial cells that migrated to cover the wound area^22^. Interestingly, these migratory cells do not undergo cell proliferation^41^, which is at least in part due to the enhanced expression of p15^ink4b ^^22,36^. These data suggest that TGFβ signaling is involved in blocking migrating cells from progressing through the cell cycle, and the findings that both TGF-βRII^22^ and p-EGFR (Fig. 1A) were upregulated in the epithelium near the wound edge introduces the possibility that the EGF and TGFβ-signaling pathways may intersect and impact one another. To investigate this potential signaling interaction, wounded corneas were excised and placed in organ culture for 18 hours in the presence of 30 μM Tyrphostin AG1478 (AG1478), an inhibitor of EGFR. No AG1478 served as a control, and the concentration was chosen based on previous data, which showed that this concentration inhibited EGFR with minimal toxicity^35,42^. After 18 hours, corneas were examined for TGF-βRII localization. In agreement with previous data^22^, TGF-βRII localization in control corneas was increased in migrating epithelial cells (Fig. 2A, arrow indicates wound edge); however, in AG1478-treated corneas, TGF-βRII localization was decreased in these cells (Fig. 2B, arrow indicates wound edge). Therefore, these data suggest that the inhibition of EGFR activation down-regulated the synthesis of TGF-βRII. Images are representative of at least 3 corneas per condition.

### Effect of EGF on TGF-βRII protein and mRNA in pHCE

**Figure 3:**
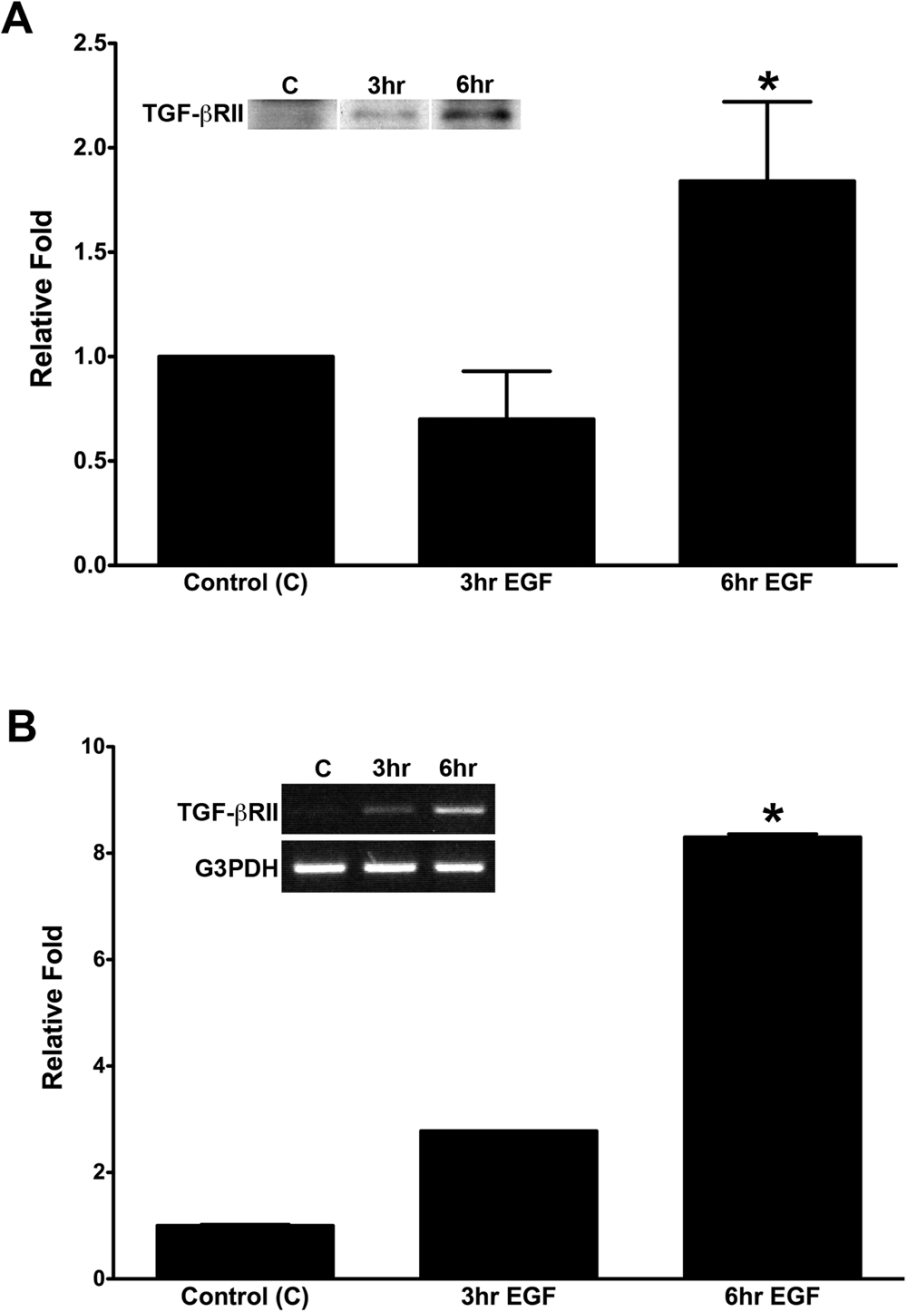
Effect of EGF on TGF-βRII in primary human corneal epithelial cells (pHCE). Protein (**A**) and mRNA (**B**) expression of TGF-βRII was examined in pHCE treated ± EGF for 3 or 6 hours (hr). Data was reported as relative fold with samples normalized to Control (C: no treatment), which was set to 1, and representative western blot (**A**) and RT-PCR (**B**) were included. G3PDH served as an internal control for RT-PCR. For both protein and mRNA, 6 hours of EGF treatment significantly increased the amount of TGF-βRII in pHCE (protein: 1.8 fold; mRNA: 8.3 fold; *p < 0.05). Original western blot and PCR gel presented are available in Supplemental Fig. S2.

To further investigate the potential interaction between the EGF and TGFβ-signaling pathways, western blot and RT-PCR analyses were used to quantify the effect of EGF on TGF-βRII protein and mRNA levels, respectively. Starved pHCE were incubated with EGF (5 ng/ml) for 3 or 6 hours, and cells in basic K-SFM medium served as Controls (C). At 3 hours, TGF-βRII protein slightly decreased (Fig. 3A; 0.7 fold; p = 0.0906) and mRNA increased (Fig. 3B; 2.78 fold; p = 0.0053), as compared with control. However, with 6 hours of EGF incubation, there was a significant increase in both the TGF-βRII protein (Fig. 3A; 1.8 fold, p = 0.0180) and mRNA (Fig. 3B; 8.3 fold, p = 0.0032). Representative western blot and RT-PCR were presented, and results were normalized to untreated controls (set to 1) and reported as relative fold ± SEM of 3 independent experiments. These data suggest that EGF acts through upregulation of both TGF-βRII mRNA and protein to propagate the signaling in pHCE.

### Effect of MEK inhibitor and EGF on TGF-βRII protein and mRNA in pHCE

**Figure 4:**
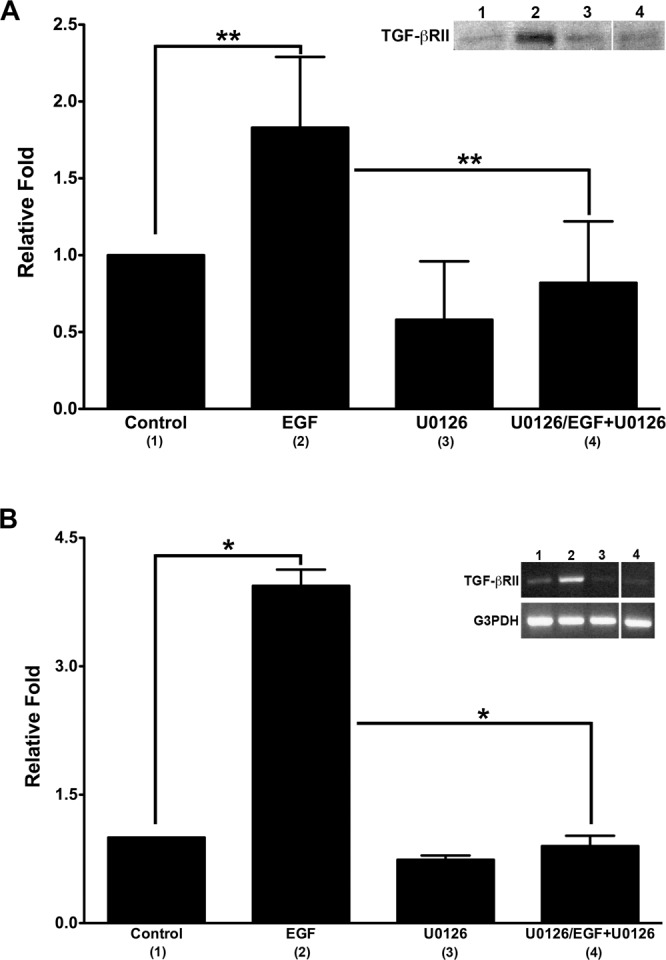
Effect of MEK inhibitor on EGF-induced TGF-βRII expression in primary human corneal epithelial cells (pHCE). Protein (**A**) and mRNA (**B**) expression of TGF-βRII was examined in pHCE treated ± EGF ± U0126 (MEK inhibitor). Data was reported as relative fold with samples normalized to Control (no treatment), which was set to 1, and representative western blot (**A**) and RT-PCR (**B**) were included. Protein and mRNA expression of TGF-βRII was significantly upregulated by EGF treatment as compared with Control (protein: **p < 0.01, 1.8 fold; mRNA: *p < 0.05, 4.0 fold), and U0126 successfully inhibited this EGF-induced upregulation of TGF-βRII expression at both protein (**A** **p < 0.01, 2.2 fold) and mRNA (**B** *p < 0.05, 4.4 fold) level. U0126 itself did not induce expression of TGF-βRII. Original western blot and PCR gel presented are available in Supplemental Fig. S3.

EGFR signaling has been reported to involve the MEK signaling pathway^43^. To continue examining EGF’s affect on TGF-βRII and to determine whether MEK inhibitor (U0126) modulates the synthesis of TGF-βRII, pHCE were serum-starved overnight and cultured for 6 hours ± EGF (5 ng/ml) ± U0126 (10 μM), and subsequently processed for western blot (Fig. 4A) and RT-PCR (Fig. 4B) to assay for TGF-βRII protein and mRNA levels, respectively. Samples that were treated with both EGF and U0126 were first pre-treated with U0126 for 15 minutes (U0126/EGF + U0126) to ensure that MEK activity was sufficiently blocked prior to growth factor treatment. Upon EGF only treatment, TGF-βRII protein (Fig. 4A; lane 2) and mRNA (Fig. 4B; lane 2) were upregulated, 1.83 (protein: p = 0.0038) and 3.94 (mRNA: p = 0.03) fold, as compared to control (Fig. 4A,B; lane 1). When pHCE were treated with U0126 only, the TGF-βRII protein and mRNA levels (Fig. 4A,B; lanes 3) remained similar to control (Fig. 4A,B; lane 1). Finally, U0126/EGF + U0126 (Fig. 4A,B; lane 4) treatment of pHCE decreased the TGF-βRII protein and mRNA expression significantly, by 55% (protein: p = 0.01) and 77% (mRNA: p = 0.04) fold respectively, as compared to EGF only (Fig. 4A,B; lane 2). Here we show that treatment with 10 μM U0126 completely blocked protein and mRNA synthesis of TGF-βRII induced by 5 ng/ml of EGF to the control level. This indicates that EGF upregulated TGF-βRII through MEK-signaling pathway in pHCE.

### Effect of EGF on p38 and ERK1/2 phosphorylation in pHCE

**Figure 5:**
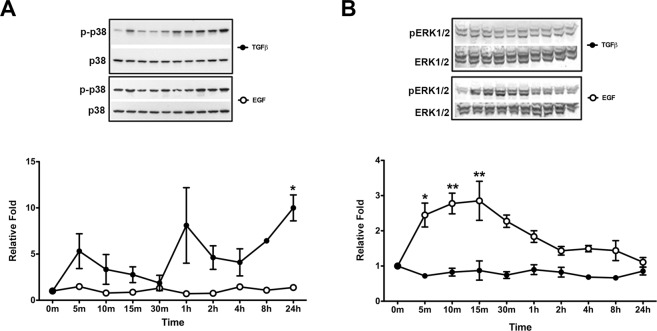
EGF and TGFβ1 time course for p38 and ERK1/2 phosphorylation. Representative western blots of pHCE incubated ± TGFβ1 or EGF for 24 hours and examined for (**A**) p-p38 and p38 or **(B**) pERK1/2 and ERK1/2. The ratios (p-p38/p38 or pERK1/2/ERK1/2) were normalized to 0 m (no treatment), which was set to 1, and reported as relative fold. (**A**) TGFβ1 stimulated the upregulation of p-p38 in a cyclical manner and was significantly (*p < 0.05) increased as compared to 0 m at 24 hrs, whereas EGF had no apparent effect on p-p38 levels. (**B**) EGF significantly stimulated the upregulation of pERK1/2 by 5 m (*p < 0.05) and 10 m (**p < 0.01), peaking at 15 m (**p < 0.05), while TGFβ1 did not activate ERK1/2. Original western blots presented are available in Supplemental Fig. S4.

In various cell types, the transmission of extracellular signals to intracellular targets is mediated by protein kinases, including the family of mitogen-activated protein kinases (MAPKs)^44,45^. MAPKs are highly conserved serine/threonine kinases that include extracellular signal-regulated kinases (ERKs), Jun NH2-terminal kinases (JNKs), and p38^MAPK^ ^46–48^. MEK is a kinase that activates MAPKs^49^. Our results (Fig. 4) indicate that EGF induces TGF-βRII expression in pHCEs through MEK. To assess if p38^MAPK^ is a target of EGF in these cells, pHCE were treated with either EGF (5 ng/ml) or TGFβ1 (2 ng/ml) for various times up to 24 hours. Protein samples were then examined for total p38 and active p38^MAPK^ (p-p38) and the ratio, p-p38/p38, was normalized to untreated pHCE (0 m), which was set to 1, and plotted. The results showed that TGFβ1 stimulated p-p38 in a cyclical manner (Fig. 5A): p-p38 was upregulated at 5 minutes, gradually decreased to a low level at 30 minutes, peaked again by 1 hour, decreased again until 4 hours, and then increased until at least 24 hours. Protein levels of p-p38 always remained higher than control levels, even at 30 minutes of treatment. Although p-p38 levels were elevated at 5 minutes (p = 0.7519) and 1 hour (p = 0.1713), the values did not reach statistical significance until 24 hours (p = 0.0387). Interestingly, when EGF was added to pHCEs, the p-p38 levels did not change significantly and hovered around baseline control levels. These data suggest that EGF has no apparent effect on p38 phosphorylation (or activity), thus indicating that EGF does not activate p38^MAPK^ in pHCEs.

Another MAPK pathway downstream of MEK, and therefore a possible target of EGF, is ERK1/2. Intriguingly, EGF and TGFβ1 exhibited opposite effects in activating ERK1/2 (Fig. 5B) than was observed with p38^MAPK^. As with p38^MAPK^, protein samples were examined for activated ERK1/2 (pERK1/2) and total ERK1/2, the ratio (pERK1/2/ERK1/2) was normalized to untreated pHCE (0 m), which was set to 1, and plotted. As indicated by the data, EGF induced a rapid upregulation of pERK1/2 at 5 minutes (2.45-fold, p = 0.0237), 10 minutes (2.78-fold, p = 0.0035), and peaked at 15 minutes (2.85-fold; p = 0.0022) before dropping to baseline control levels at 24 hours. TGFβ1, on the other hand, did not induce any significant difference in pERK1/2 as compared to control over the course of the 24-hour culture period.

**Figure 6:**
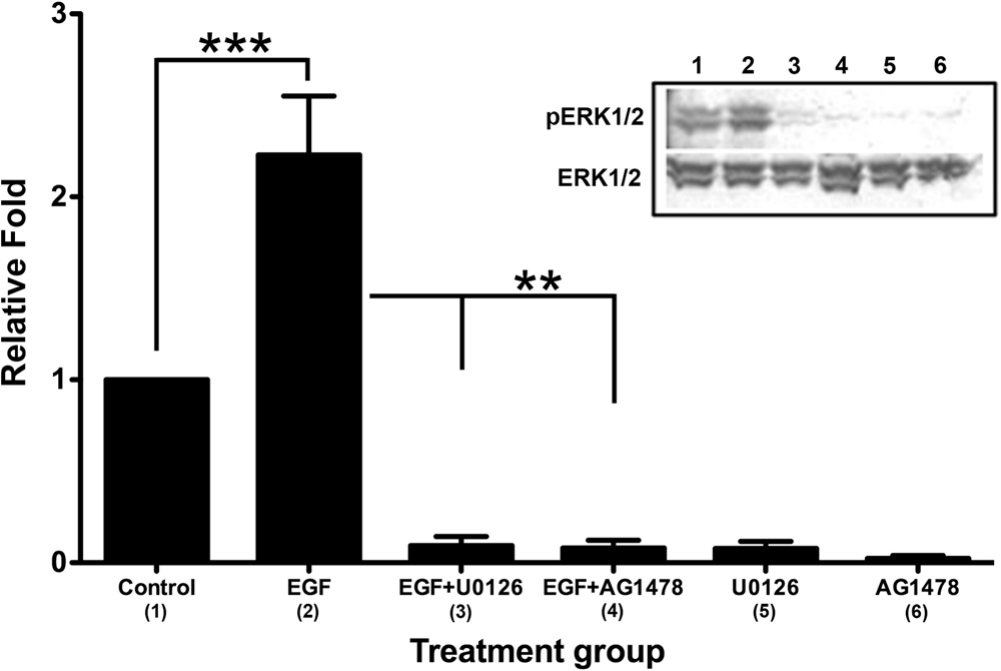
Effect of MEK and EGFR inhibitors on EGF-induced activation of ERK1/2 in primary human corneal epithelial cells (pHCE). Representative western blot of pHCE incubated for 15 minutes ± EGF ± U0126 (MEK inhibitor) or AG1478 (EGFR inhibitor) and examined for pERK1/2 and ERK1/2. The ratio (pERK1/2/ERK1/2) was normalized to Control (no treatment), which was set to 1, and reported as relative fold. EGF significantly (***p < 0.001) upregulated pERK1/2 as compared with control. EGF-induced phosphorylation of ERK1/2 was significantly (**p < 0.01) blocked by both U0126 and AG1478 + EGF. Neither U0126 nor AG1478 stimulated pERK1/2. Original western blot presented is available in Supplemental Fig. S5.

To evaluate whether EGF’s activation of ERK1/2 was EGFR-signaling dependent, pHCE were treated with EGF ± AG1478 ± U0126 for 15 minutes, since this was the peak for ERK1/2 activation observed in Fig. 5B. Protein samples were examined by western blot for pERK1/2 and ERK1/2, the ratio (pERK1/2/ERK1/2) was normalized to untreated controls (Control), which was set to 1, and the values were plotted. In agreement with the data from Fig. 5B, EGF significantly upregulated pERK1/2 as compared to control (Fig. 6, lanes 1 and 2; p = 0.0004). Both U0126 and AG1478 potently blocked EGF-induced pERK1/2 to levels below baseline control (Fig. 6, lanes 3 and 4; p = 0.0049 and 0.0043, respectively). Addition of U0126 or AG1478 alone to pHCE for 15 minutes also significantly reduced pERK1/2 below baseline (Fig. 6, lanes 5 and 6; p = 0.0043 and 0.0027, respectively). These data indicate that the activation of ERK1/2 by EGF in pHCE requires both the MEK- and EGFR-signaling cascade. Moreover, it is interesting to note that despite serum-starving pHCE overnight, the cells express basal levels of ERK1/2 activation that can be significantly blocked by either U0126 or AG1478. This suggests that the basal levels of pERK1/2 in our untreated control are EGFR and MEK dependent.

## Discussion

A complex signaling network of growth factors and cytokines orchestrates cell migration, proliferation, differentiation, apoptosis, and wound healing in different cell types, with various stimuli activating divergent signaling pathways and inducing distinct cellular responses^50^. TGFβ and EGF are among these stimuli, with TGFβ playing a critical role in controlling cellular growth and ECM deposition^51,52^, and EGF regulating cell proliferation, differentiation, and survival^53^. We have shown that both EGFR and TGF-βRII were activated in the corneal epithelium upon wounding^22,35^, and despite EGF and TGFβ’s apparently distinct roles on corneal epithelial proliferation^54,55^, it is reasonable to speculate that EGF- and TGFβ-signaling pathways may converge to elicit cellular effects during corneal wound healing.

In the present study, we investigated the regulation of the TGFβ-signaling pathway by EGF by observing the synthesis of TGF-βRII at both the mRNA and protein levels in corneal epithelial tissue using a rat corneal debridement wound-healing model, *in vivo* and organ culture, and pHCE. Consistent with previous studies^35^, we found that EGF was involved in the corneal epithelial wound-healing process (Fig. 1), and EGFR was activated as early as 15 minutes post-wounding with localization mostly at the leading edge, especially in the basal epithelial aspect adjacent to the basement membrane, but also extending all the way to the peripheral cornea (Fig. 1A.a–c). This time point turned out to be the peak of EGFR activation in wounded epithelium (Fig. 1B). In addition, TGF-βRII localization was upregulated in the migrating epithelial cells after a corneal epithelial wound (Fig. 2A), and was inhibited in the presence of the EGFR inhibitor AG1478 (Fig. 2B). Finally, the cultured pHCE studies showed that with 6 hours of EGF stimulation, the amount of TGF-βRII protein and mRNA in pHCEs was significantly increased (Fig. 3). These data indicate that EGF stimulated TGF-βRII synthesis in the corneal epithelial cells, and inhibition of EGFR activation blocked the synthesis of TGF-βRII in cells at the leading edge. Therefore, the EGF and TGFβ-signaling pathways interact.

From this data and what is known about EGF, it appears that EGF has a dual effect on TGFβ signaling. For instance, both EGF and TGFβ1 stimulate TGF-βRII expression in corneal epithelial cells and human dermal fibroblasts^56^, which is necessary for propagation of signaling. Also, it has been reported that EGF synergizes with TGFβ1 in rabbit corneal keratocytes on the migration of myofibroblasts^37^. However, in human corneal epithelial cells, EGF attenuates TGFβ1-induced p15^INK4b^ expression by 51.6%^22^. Our previous studies have shown that p15^INK4b^ stimulates pHCE migration^36^ and EGF inhibits TGFβ1-induced p15^INK4b ^^22,36^, thereby abrogating the stimulating effect of TGFβ1 on pHCE migration. Therefore, EGF synergizes or antagonizes TGFβ signaling, depending on the target protein of TGFβ1 and cell type. In addition, based on the *in vivo* data that EGFR peaks at 15 minutes (Fig. 1) and previous data that TGF-βRI and II peak at 16 hours after a corneal epithelial wound^22^, this dual regulation of EGF on TGFβ signaling most likely is under temporal and spatial control. Therefore, we postulate that EGF signaling dominates early events, such as corneal epithelial proliferation and survival because of the low level of TGF-βRII in the epithelial cells; however, over time, EGFR decreases and TGF-βRII increases in these cells, suggesting that TGFβ signaling will dominate later events. This would allow cells at the leading edge to be stimulated to migrate, but blocked from proliferating, perhaps resulting in optimized migration. Of particular interest is how the opposing effects of EGF on TGFβ signaling fits into the spatio-temporal stages of corneal epithelial wound healing. Our future studies will explore the functional importance of the ability of EGF to both inhibit and synergize with TGFβ, thereby establishing a link between the opposing roles and the morphological and biochemical events (migration, mitosis and differentiation) during corneal wound healing.

Our finding that EGF alone is able to upregulate the expression of TGF-βRII raises interesting questions regarding the downstream interactions between EGFR and TGFβ-associated intracellular signaling pathways. The MEK pathway is typically involved in activation of EGF signaling. Therefore, in the present study, we explored the role of the MEK pathway in EGF-induced upregulation of TGF-βRII by using the MEK inhibitor, U0126. As seen in Fig. 4, addition of U0126 completely blocked the protein (Fig. 4A) and mRNA (Fig. 4B) synthesis of TGF-βRII induced by EGF to control levels, indicating that EGF crosstalks with TGFβ signaling through MEK-signaling. It is well known that p38^MAPK^ is a downstream signaling pathway of TGFβ^57^. However, as seen in Fig. 5A, EGF failed to phosphorylate p38^MAPK^ in pHCE. Therefore, we postulate that p38^MAPK^ is not involved in EGF’s regulation of TGFβ signaling in pHCE. Interestingly, we found that the MEK-signaling pathway, ERK1/2, was potently activated by EGF, peaking at 15 minutes, and was not activated by TGFβ stimulation (Fig. 5B). These results suggest that EGF-induced upregulation of TGF-βRII may be acting through the ERK1/2-signaling pathway. It appears that the mechanisms underlying EGF-induced TGF-βRII may vary in different tissues. For example, human dermal fibroblast research showed that EGF upregulated TGF-βRII through the p38^MAPK^-signaling pathway independent of the MEK/ERK-signaling pathway^56^. In addition, it was also reported that EGF upregulated TGF-βRII via PI-3 kinase in rabbit corneal stromal cells^37^. Hence, EGF may regulate TGFβ signaling via different mechanisms in different cell types. Future studies exploring the role of PI-3 kinase may be a potential signaling pathway for our model.

TGF-βRII is typically activated by a TGFβ ligand, causing TGF-βRII to then associate with and activate TGF-βRI to subsequently control various downstream signaling pathways including the canonical Smad and non-canonical signaling pathways^23^. Our previous study showed that EGF was unable to stimulate the expression of TGF-βRI^22^. This raises interesting questions regarding the extent to which EGF alone is able to activate aspects of the downstream TGFβ-signaling cascade and its role in wound healing and fibrosis. For example, can EGF itself also phosphorylate the canonical TGFβ/Smad proteins (Smad2/3)? In a rabbit model of stromal wound healing, EGF was shown to exhibit similar functional activity to TGFβ. He and Bazan showed that EGF was able to stimulate the differentiation of corneal keratocytes into alpha-smooth muscle actin-positive myofibroblasts^37^. Treatment with TGFβ1 induced 12% of the cells to differentiate into myofibroblasts; however, in the presence of EGF, this increased to 90% and further enhanced cell migration compared to either growth factor alone^37^. These results indicate that not only can EGF exhibit similar capabilities as TGFβ; it can also augment the effects of TGFβ in stromal wound healing. This poses interesting questions as to whether these findings translate to corneal epithelial wound healing: does co-treatment with EGF and TGFβ result in accelerated migration and wound healing in corneal epithelial cells compared to either growth factor alone?

Aside from EGF, several other ligands belong to the EGF family of growth factors including heparin-binding EGF-like growth factor (HB-EGF), transforming growth factor-alpha (TGFα), betacellulin (BTC), amphiregulin (AR) and epiregulin (EPR)^2^. HB-EGF enhances corneal epithelial wound healing and keratinocyte-specific HB-EGF deficient mice showed delayed healing in the corneal epithelium^3^. In fact, HB-EGF has been shown to stimulate better wound healing responses than EGF^58^. Peterson *et al*.^39^ explored the role of all six endogenous EGFR ligands and showed that betacellulin was the most effective at promoting wound healing *in vitro*. In contrast, only EGF was able to promote wound healing *in vivo*^39^. This poses interesting questions for future investigation regarding whether our finding that EGF can upregulate TGF-βRII is specific to EGF itself or if this mechanism translates to other members of the EGF family.

In conclusion, we showed that both EGF and TGFβ expression are stimulated upon wounding of the corneal epithelium. EGF stimulates TGF-βRII upregulation through MEK/ERK; however, it is not p38^MAPK^ dependent. Our findings enhance the current understanding of the integrated activity of EGF and TGFβ pathways during corneal wound repair. A few clinical studies have demonstrated that EGF eye drops accelerated healing of epithelial defects^15,17,59^, and our study provides insights into the molecular mechanisms underpinning the efficacy of EGF. Continual pursuits in extending our current understanding of the underlying molecular mechanisms of corneal wound repair will enable us to improve drug design to selectively modulate specific phases of the corneal wound healing process, thereby accelerating wound closure, minimizing scarring, and preserving the normal corneal architecture.

## Methods

### Debridement wound

As previously described, a 3-mm debridement wound was made on the central cornea of Sprague-Dawley rats^22,60^. The corneas were either allowed to heal *in vivo* or were excised and placed in organ culture^61^. For the *in vivo* experiments, corneas were allowed to heal for up to 24 hours. At the appropriate time, the corneas were processed for either indirect-immunofluorescence (IF) by enucleating and freezing in OCT or western blotting (WB) by scraping and flash freezing the corneal epithelium. For the organ culture experiments, corneas were allowed to heal for 18 hours in EMEM medium (ATCC; Manassas, VA) ± 30 μm AG1478, an inhibitor of EGFR (Sigma-Aldrich; St. Louis, MO). The corneas were then frozen in OCT for IF^62^. All protocols in this study conformed to the standards of the ARVO Statement for the Use of Animals in Ophthalmic Care and Vision Research and were approved by the Schepens Eye Research Institute/Mass. Eye and Ear Animal Care and Use Committee.

### Indirect-immunofluorescence (IF) microscopy

IF was performed using 6-micron cryostat sections, as previously published^61^. Sections were incubated at room temperature for 1 hour with primary antibodies against p-EGFR (Cell Signaling Technology; Danvers, MA) or TGF-βRII (Santa Cruz Biotechnology; Santa Cruz, CA), followed by a 1-hour incubation of corresponding secondary antibody (Jackson ImmunoResearch; West Grove, PA). Coverslips were mounted with vectashield mounting media (Vector Laboratories; Burlingame, CA) ± Propidium Iodide, a nuclear counterstain. Negative controls where the primary antibody was omitted were run with every experiment. The sections were viewed and photographed under a Nikon Eclipse E800 microscope equipped with a Spot Camera (Nikon; Melville, NY).

### Isolation of primary culture of human corneal cells

Primary human corneal epithelial cells (pHCEs) were isolated from human limbal rims^63^ obtained from Dr. Repoza of Ophthalmic Consultants of Boston (Boston, MA). These limbal rims were excess corneal tissue that otherwise would have been discarded after corneal transplantation, no identifying information was obtained. Procedures/methods used in these studies adhered to the tenets of the Declaration of Helsinki, and the Schepens Eye Research Institute IRB deemed this research to be exempt. In brief, limbal rims were rinsed with Dulbecco’s-PBS (D-PBS—without calcium or magnesium: Invitrogen/Life Technologies; Grand Island, NY) containing 20 μg/ml of Gentamicin (Invitrogen) for 2–3 minutes before cutting into 8 pieces of equal size. These small pieces of tissue were treated with dispase solution (25 Caseinolytic Units/ml: Becton Dickinson Labware; Franklin Lakes, NJ) in Hanks’ Balanced Salt Solution (Invitrogen) with 5 μg/ml of Gentamycin for 18–24 hours at 4 °C. After incubation in dispase, the epithelial layer was lifted from the stroma and digested in trypsin-EDTA solution (Invitrogen) for 5 minutes at 37 °C. During this time, the mixture of tissue and trypsin was aspirated with a small pipette in order to dissociate the cells. After 5 minutes, an equal volume of 10% FBS (Gemini Bio-products, Inc.; Calabasas, CA) in D-PBS was added to the tissue and trypsin mixture in order to neutralize the trypsin. Cells were pelleted at 1000 rpm for 5 minutes, resuspended in Keratinocyte-SFM (K-SFM: Invitrogen), and seeded onto a flask coated with FNC Coating mix (AthenaES; Baltimore, MD). Medium was changed every other day until cells reached 70–80% confluence.

### Cell Culture

Primary HCEs (pHCEs) were plated in T25 flasks, grown to 70–80% confluence and starved overnight by incubating cells in basic K-SFM (without EGF and bovine pituitary extract [BPE] supplements). After starving the cells, they were treated with 2 ng/ml TGF-β1 (R&D Systems; Minneapolis, MN), 5 ng/ml EGF (R&D Systems), 10 μM MEK inhibitor (U0126; Promega; Madison, WI), or 30 μM EGFR inhibitor (Tyrphostin AG1478: Selleck Chemical LLC/Thermo Fisher Scientific; Pittsburgh, PA) alone or in combination for specified times at 37 °C in a humidified 5% CO_2_ incubator. Cells in basic K-SFM served as controls. Cells were harvested for western blot (WB) or reverse transcriptase-polymerase chain reaction (RT-PCR).

### Western Blot (WB) Analysis

For WB assays, the harvested cells or epithelial tissue were lysed with RIPA buffer^35^, and the protein concentration was determined using a protein assay kit (Bio-Rad Protein Assay; Hercules, CA). Equal amounts of protein from cell extracts were loaded and electrophoresed on a 4–20% gradient Tris-glycine gel (Invitrogen). The protein was electrophoretically transferred to a PVDF membrane (Millipore; Burlington, MA), and the transfer was confirmed by staining the membrane with 0.1% Ponceau S solution (Sigma-Aldrich). The membrane was then blocked for 1-hour in Blotto blocking reagent (5% dry milk in TTBS [tris buffered saline +0.1% Tween 20]). After blocking, the membrane was incubated with anti-p38 (Santa Cruz), p-p38 (Santa Cruz), ERK1/2 (Cell Signaling), pERK1/2 (Cell Signaling), TGF-βRII, or p-EGFR (Upstate Biotechnology; Lake Placid, NY) in Blotto for 1 hour, washed briefly, and incubated for an additional hour with corresponding peroxidase-conjugated (Santa Cruz Biotechnology) or fluorescently labeled (IRDye 680RD or 800CW: Li-Cor Biosciences; Lincoln, NE) secondary antibody. For the membranes treated with peroxidase-conjugated secondary antibody, they were then soaked in chemiluminescent substrate (SuperSignal Substrate; Thermo Scientific/Pierce Biotechnolgy; Rockford, IL) for 5 minutes and exposed to x-ray film. The film was developed and the band intensities quantified using ImageJ software (National Institutes of Health; Bethesda, MD: http://rsb.info.nih.gov/ij/). For the membranes treated with fluorescently labeled secondary antibody, they were viewed on an infrared imaging system (Odyssey: Li-Cor; Lincoln, Nebraska), and the protein bands were analyzed with the Li-Cor Odyssey Image Studio Ver2.1. β-actin was used as an internal control.

### Reverse Transcriptase-Polymerase Chain Reaction (RT-PCR) Assay

Total RNA was isolated from the treated pHCEs using TrIzol Reagent (Gibco BRL/Thermo Fisher Scientific). RT-PCR was performed as previously described^64^, with specific primers for human TGF-βRII and G3PDH (Table 1), which served as an internal control for cDNA quantity and quality. The PCR products were run on a 1.5%-agarose gel containing 0.5 μg/ml ethidium bromide. The gel was photographed with a Bio-Rad Gel Doc 2000, and the band intensities were quantified using ImageJ software. Samples with no cDNA were also amplified and served as negative controls.

**Table 1:** Oligonucleotide primer sequences.

Primer	Oligonucleotide sequence	Annealing Temp	Fragment size	Reference
TGF-βRII	5′-TGTGAGAAGCCCGCAGGAAGTC-3′ 5′-GGACATCTTCTCTGACATCAA-3′	50 °C	650 bp	Joyce and Zieske^9^
G3PDH	5′-ACCACAGTCCATGCCATCAC-3′ 5′-TCCACCACCCTGTTGCTGTA-3′	52 °C	452 bp	Clonetech Laboratories, Palo Alto, CA, U.S.A

### Statistical Analysis

Experiments were replicated at least three times, and statistical significance and p values were determined (GraphPad Prism v.5a: Graphpad Software Inc.; La Jolla, CA) with either Student’s t-test or ANOVA followed by Tukey’s post hoc test. P values were reported in figures as *p < 0.05, **p < 0.01, and ***p < 0.001.

## Supplementary information

Supplementary Information

## Data Availability

Original western blots and PCR gels that were presented in the figures are presented in the Supplemental data. All other data is available upon request.
