# Supplementary material for: Epidermal Growth Factor Stimulates Transforming Growth Factor-Beta Receptor Type II Expression In Corneal Epithelial Cells
Source: Sci Rep. 2019 May 30;9:8079. doi: 10.1038/s41598-019-42969-2 (PMC6542834; doi:10.1038/s41598-019-42969-2)
Supplement: Supplementary file 1 — Supplementary Information [file 41598_2019_42969_MOESM1_ESM.docx]

# Epidermal Growth Factor Stimulates Transforming Growth Factor-Beta Receptor Type II Expression In Corneal Epithelial Cells

Daisy Y. Shu, Audrey E.K. Hutcheon, James D. Zieske, and Xiaoqing Guo

**SUPPLEMENTARY FIGURES**

**Supplementary Figure S1**

| 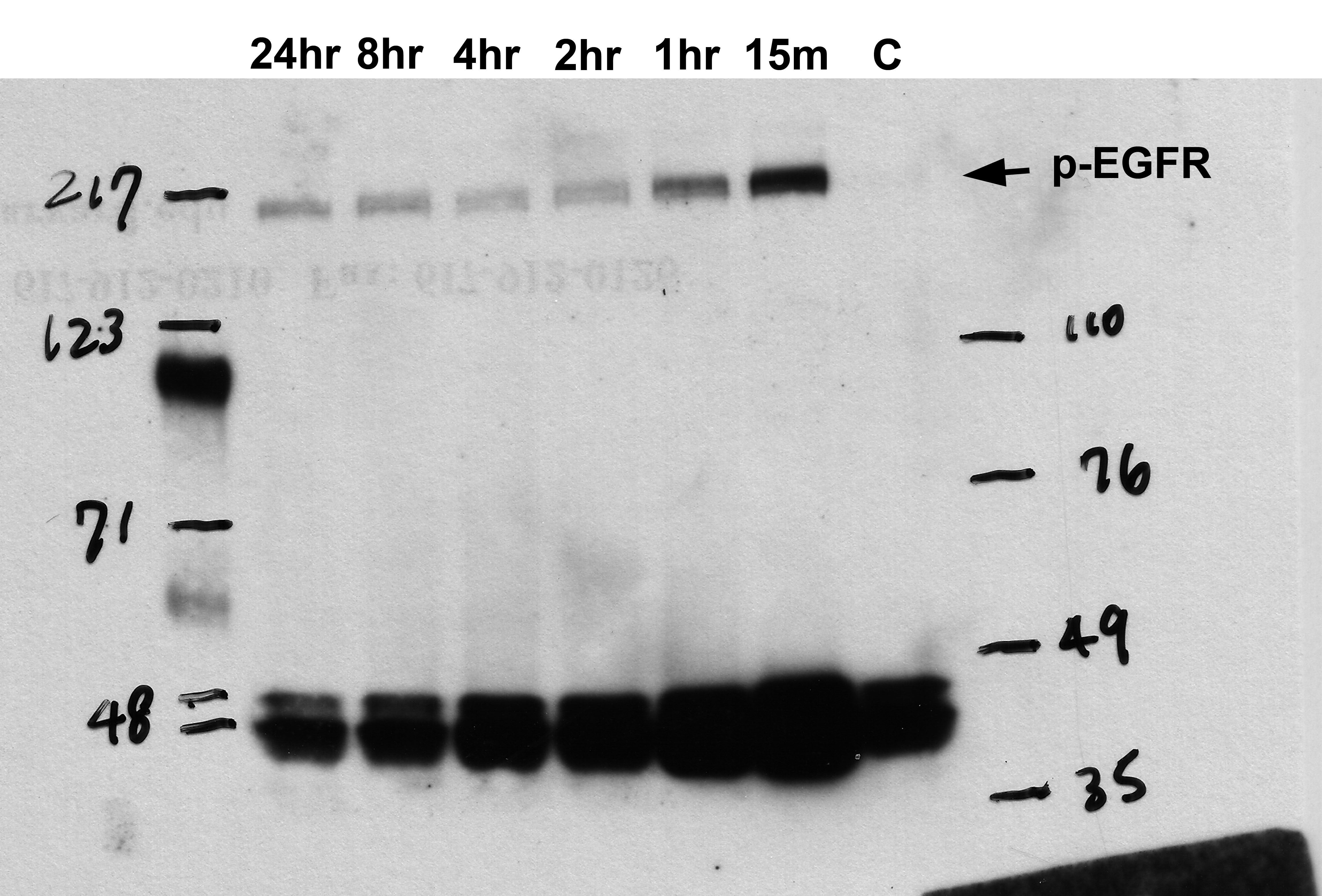 | **Supplementary Figure S1:** Original whole western blot shown. For the final figure, photoshop was used to flip the western blot horizontally and crop the horizontal line of bands indicated by arrow and p-EGFR on the right. The levels were adjusted so that the bands were more apparent. The whole horizontal line of bands was adjusted at the same time. They were then placed in the Figure 1. |
| --- | --- |

**Supplementary Figure S2:**

| 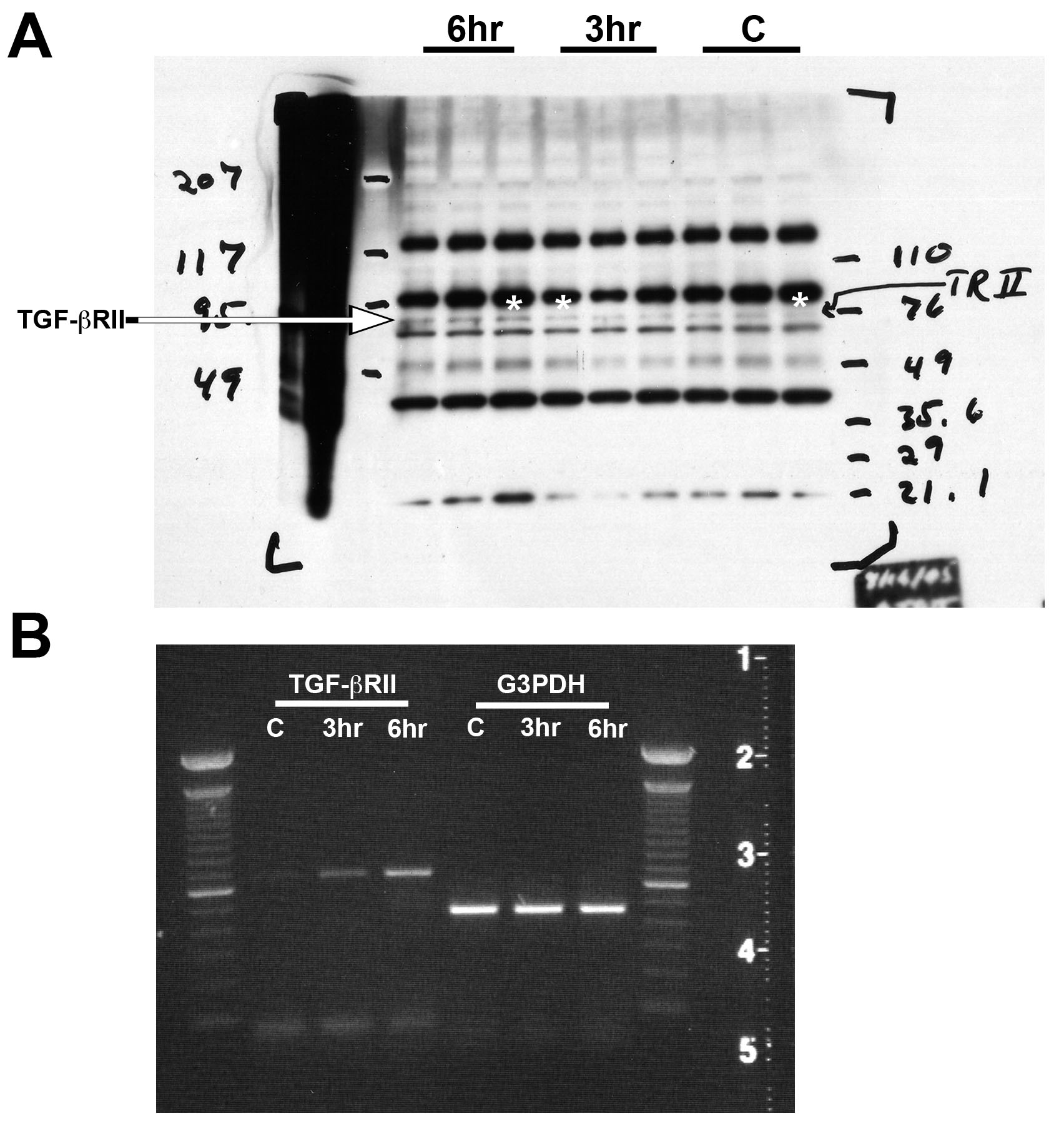 | **Supplementary Figure S2A:**Original whole western blot shown. For the final figure, photoshop was used to flip the western blot horizontally, crop the horizontal line of bands indicated by arrow and TGF-βRII, and adjust the levels so that the bands were more apparent. The whole horizontal line of bands was adjusted at the same time. Samples were loaded and run in triplicate blot. A representative lane was chosen from each sample set (*) and made placed into Figure 3A.  **Supplementary Figure S2B:**Original whole PCR gel shown. For the final figure, photoshop was used to adjust the levels so that the bands were more apparent. The whole blot was adjusted at the same time. Then, the TGF-βRII and G3PDH bands were cropped from the image and placed in the Figure 3B. |
| --- | --- |

**Supplementary Figure S3:**

| 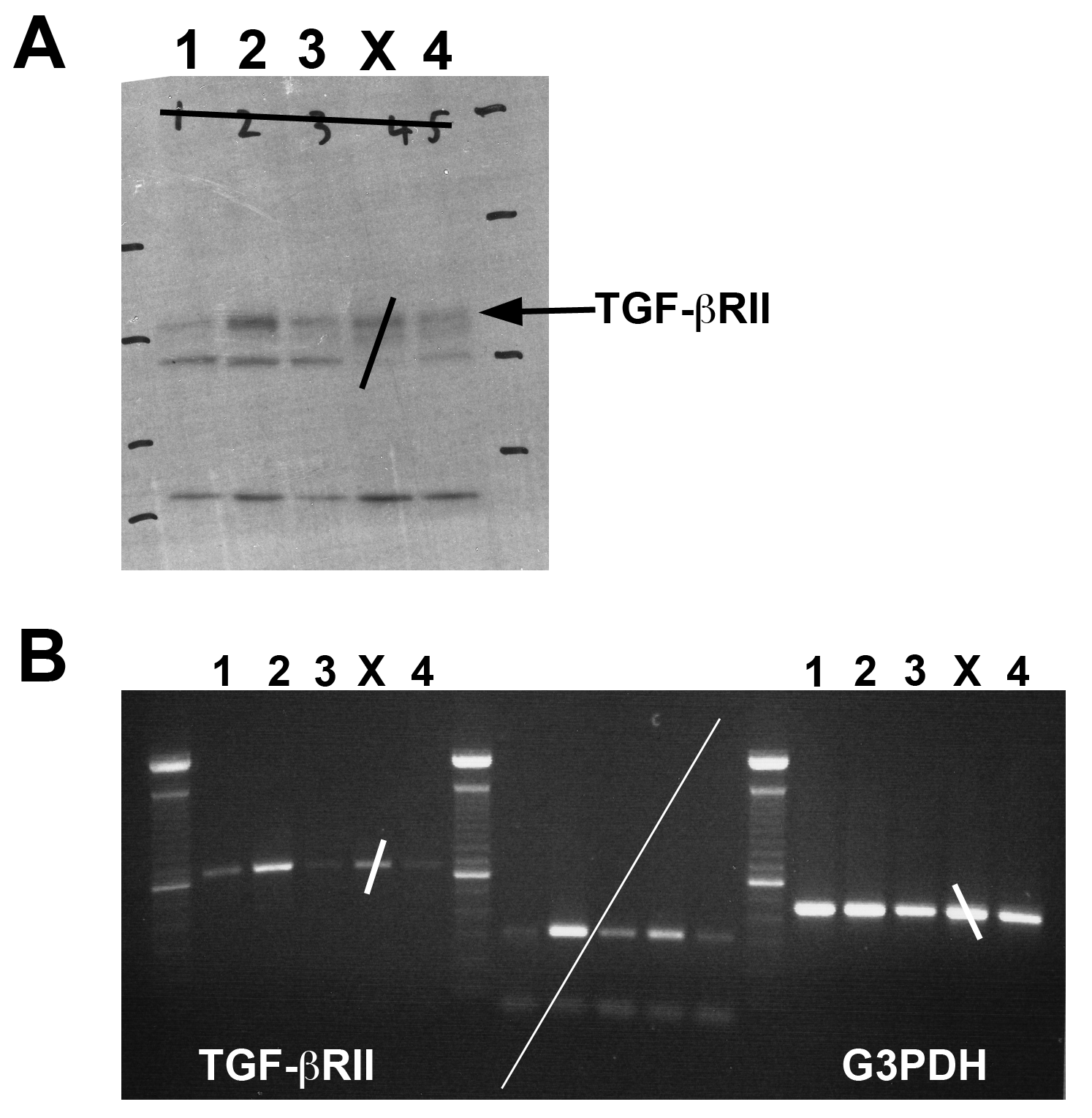 | **Supplementary Figure S3A:**Original whole western blot shown. For the final figure, photoshop was used to crop the topmost horizontal line of bands (arrow), and adjust the levels so that the bands were more apparent. The whole horizontal line of bands was adjusted at the same time. The band in lane 4 was removed and the rest of the bands were placed in Figure 4A.  **Supplementary Figure S3B:**Original whole PCR gel shown. For the final figure, photoshop was used to adjust the levels so that the bands were more apparent. The whole blot was adjusted at the same time. Then, the TGF-βRII and G3PDH bands were cropped from the image and placed together. The fourth lane from each (X) was removed, and the final image was placed in Figure 4B. |
| --- | --- |

**Supplementary Figure S4:**

| 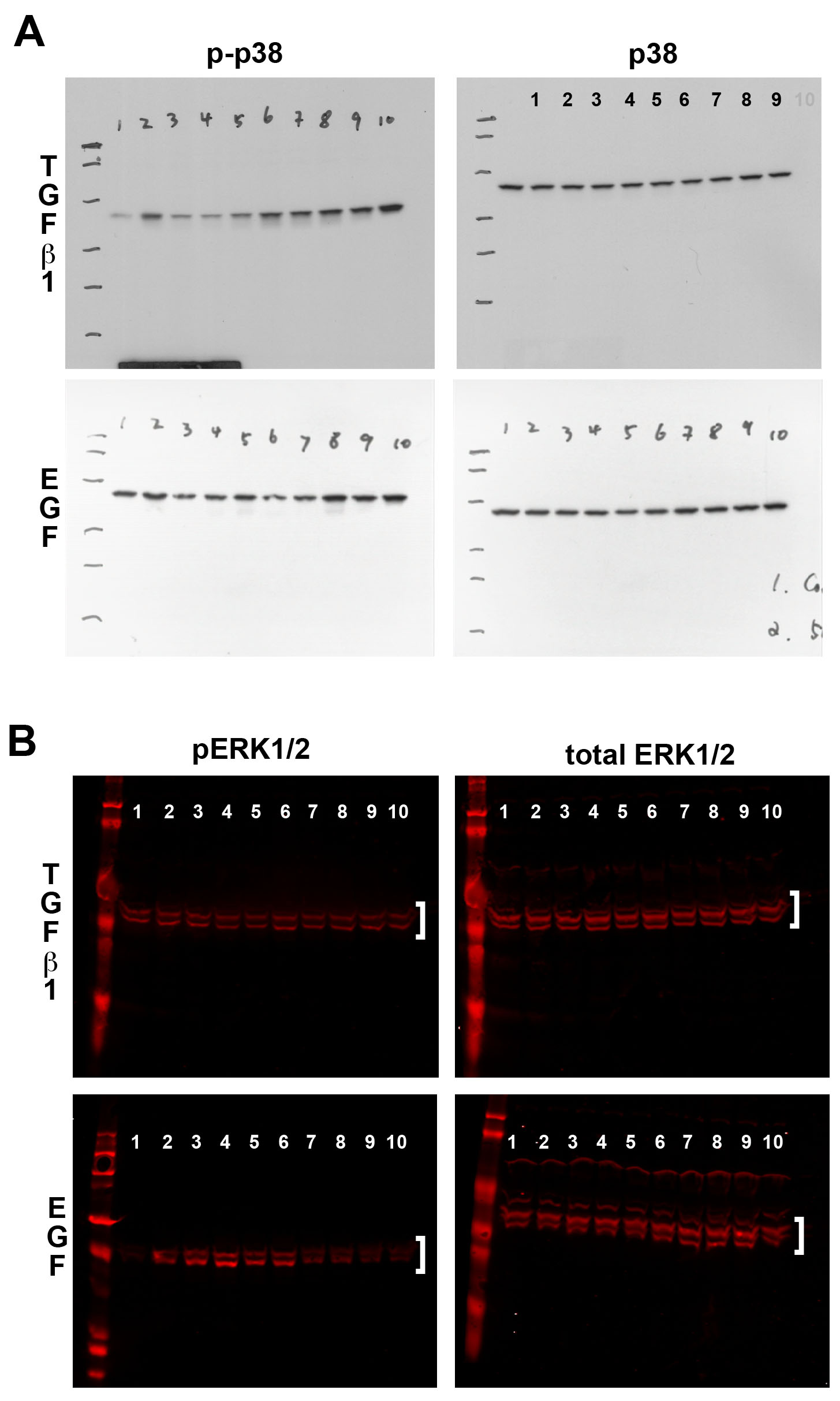 | **Supplementary Figure S4A:** Original whole western blot shown. For the final figure, photoshop was used to crop the horizontal line of bands and adjust the levels so that the bands were more apparent. The whole horizontal line of bands was adjusted at the same time and placed in Figure 5A.  **Supplementary Figure S4B:** Original whole western blot shown. For the final figure, photoshop was used to change the mode from RGB to grayscale, invert, crop the horizontal line of bands (bracket), and adjust levels so that the bands were more apparent. The whole horizontal line of bands was adjusted at the same time and placed in Figure 5B. |
| --- | --- |

**Supplementary Figure S5:**

| 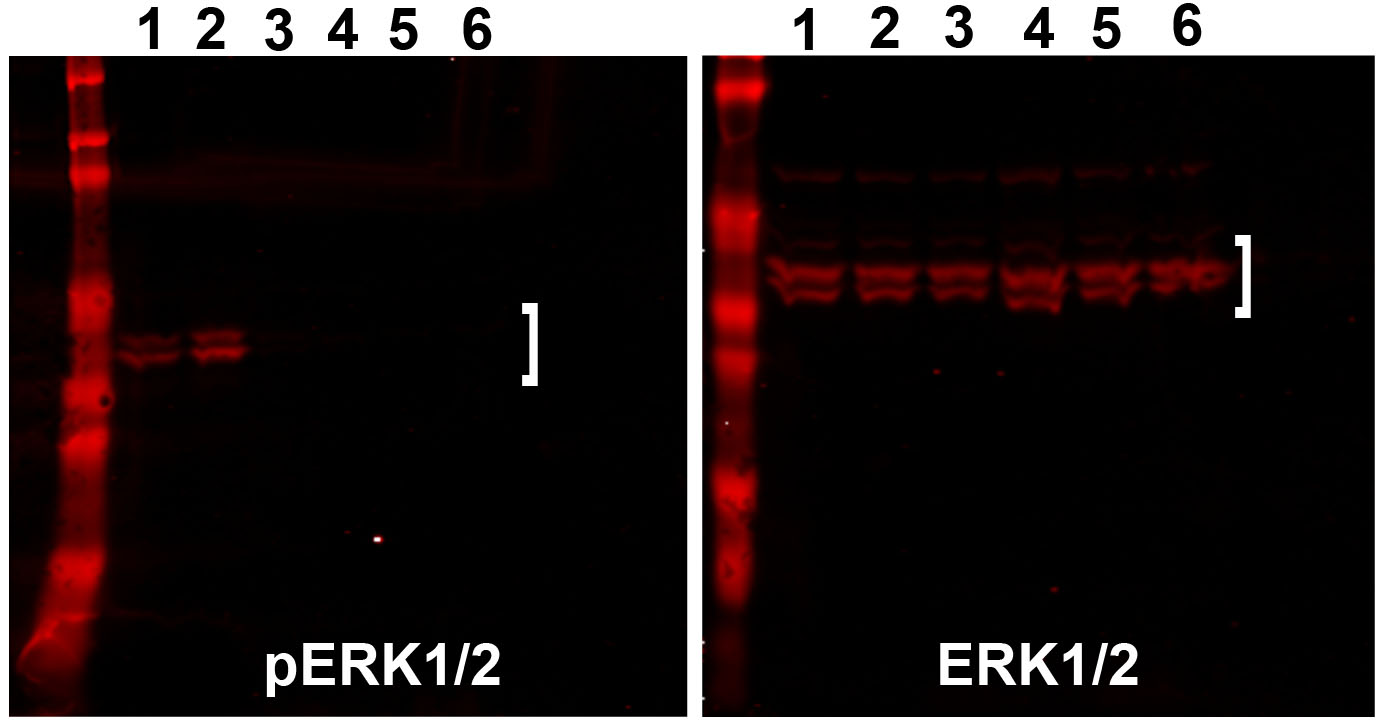 | **Supplementary Figure S5:** Original whole western blot shown. For the final figure, photoshop was used to change the mode from RGB to grayscale, invert, crop the horizontal line of bands (bracket), and adjust levels so that the bands were more apparent. The whole horizontal line of bands was adjusted at the same time and placed in Figure 6. |
| --- | --- |
